# Buffalo State Lunatic Asylum

**Published:** 1870-05

**Authors:** 


### Buffalo State Lunatic Asylum.

The managers of the Buffalo State Lunatic Asylum, appointed by the Governor, consist of the following gentlemen:

Dr. John P. Gray, Utica Asylum.
Hon. Augustus Frank, Wayne County.
Hon. Horatio Morris, Chautauqua County.
Dr. William P. Gould, Niagara County.
Hon. Asher P. Nichols, Buffalo.
Hon. Dennis Bowen, "
Hon. A. P. Laning, "
Joseph Warren, Esq., "
George R. Yaw, Esq., "
Prof. James P. White, M. D.,"

The first meeting will be held at the Tifft House, in Buffalo, Thursday, May 26, at 11 A. M.

